# Advances in Nanocomposite Membranes

**DOI:** 10.3390/membranes11030158

**Published:** 2021-02-25

**Authors:** Pei Sean Goh, Ahmad Fauzi Ismail

**Affiliations:** Advanced Membrane Technology Research Centre, School of Chemical and Energy Engineering, Universiti Teknologi Malaysia, Johor Bahru 81310, Malaysia; afauzi@utm.my

The design and synthesis of functional nanomaterials have been extensively explored over the last decade, primarily due to their exceptional physico-chemical properties. Various nanomaterials have been used for the development of nanocomposite membranes due to the vast potential in harnessing their compositional and structural advantages. The desired physical and chemical properties endowed by nanomaterials at the molecular level can potentially address the limitations and bottlenecks of conventionally used polymeric and ceramic membranes. The development of nanocomposite membranes involves multidisciplinary strategies that include the innovative design and applications of nanocomposite membranes. The structural and separation properties of membranes can be feasibly tailored and fine-tuned through the introduction of various natural and engineered nanomaterials. When introduced on a surface or within a matrix of membranes, nanomaterials characterized by different dimensional and structural properties render synergetic effects that are unmet by conventionally fabricated membranes.

The Special Issue of “Advances in Nanocomposite Membranes” aims to bring together membrane scientists and engineers to look into multiple aspects associated with the development of state-of-the-art nanocomposite membranes. This Special Issue covers important topics associated to the design and fabrication of nanocomposite membranes, their functionalization and modifications approaches, and the applications of these advanced membranes in niche separation processes, including gas separation, wastewater treatment, and desalination. In total, nine contributions, comprising seven research articles and two reviews, have been successfully compiled in this Special Issue. This collection of research articles and reviews focuses on the synthesis, design, optimization, and applications of advanced nanocomposite membranes, and it also looks into the recent advancements and future trends of this realm. 

Metal oxide-based nanomaterials such as titania (TiO_2_) and carbon-based nanomaterials such as graphene oxide (GO) have been extensively used for the development of nanocomposite membranes due to their desired surface hydrophilicity, as well as their textural and structural properties. Based on three types of TiO_2_-incorporated polyvinylidene fluoride (PVDF) membranes, Teow et al. [1] performed a correlation study between membrane surface morphologies and their fouling behaviors in natural organic matter-containing surface water. The nanocomposite membrane incorporated with X500 TiO_2_ exhibited promising anti-fouling and UV-defouling properties, primarily ascribed to its smooth surface enriched with abundant reactive surface groups. By coupling sol–gel and modified phase inversion techniques, Peixoto et al. [2] prepared a cellulose acetate (CA)-based nanocomposite incorporated with silica (SiO_2_) and TiO_2_. In the former nanocomposite membrane, the covalently bound TiO_2_–SiO_2_ was bound to CA through the interaction between SiO_2_ and CA, while in the latter, the TiO_2_ was directly bound to CA. The binding site significantly affected the permeability of the resultant nanocomposites, where the introduction of single TiO_2_ rendered an improvement of hydraulic permeability by more than two folds when compared to the neat CA membrane. On the other hand, the presence of SiO_2_ resulted in a neglected change in membrane permeability. 

Alnoor et al. [3] modified the surface of GO and reduced graphene oxide (rGO)-deposited polyethersulfone (PES) microfiltration membranes with low-pressure plasma treatment. It was observed that the plasma treatment with a duration of two minutes enhanced the interfacial interaction between the GO or rGO layer and the PES membrane surface. The improved adhesion and stability of the deposited nanomaterial layers were evidenced from their increased potassium chloride ion rejection compared to neat and nanocomposite membranes treated with different plasma treatment conditions. Compared to GO, the deposition of rGO helped to minimize the occurrence of micro- or nano-cracks on the membrane surface, hence further improving the ion rejection to 94%. Roslan et al. [4] proposed a feasible approach to introduce a coating of GO on polysulfone (PSF) hollow fiber membranes. A nanocomposite gas separation membrane consisting of a GO nanosheet, a polyether block amide (Pebax), a polydimethylsiloxane (PDMS) gutter layer, and PSF was developed. Their findings suggested that the introduction of 0.8 wt% GO into the Pebax selective layer resulted in the best performing multilayered nanocomposite membrane, which exhibited 56.1% and 20.9% enhancements in the CO_2_/CH_4_ and O_2_/N_2_ gas pair selectivities, respectively, when compared to the neat membranes. 

Nanocomposite membranes incorporated with bi- or tri-metallic nanoparticles were developed by Ndlwana et al. [5] by blending PES with hyperbranched polyethyleneimine (HPEI) supported bi- (Pd@Fe@HPEI) or tri-metallic (Pd@FeAg@HPEI) nanoparticles. The nanocomposite membranes demonstrated increased surface hydrophilicity, textural properties, and pure water permeability. The desired characteristics rendered by the nanomaterials also helped to reduce protein fouling. Quantum dots are semiconductor nanocrystals that show exceptional properties due to quantum mechanics. Gan et al. [6] incorporated lemon-derived, carbon quantum dot (CQD)-grafted, silver nanoparticles (Ag) (Ag/CQDs) into a PSF membrane for dye removal. Hydrothermal treatment was employed to carbonize the pulp-free lemon juice into a CQD solution. The findings indicated that the incorporation of 0.5 wt% Ag/CQDs significant improved the pure water permeability and dye rejection by 169% and 92%, respectively, compared to the neat PSF membranes. The nanocomposite membrane also demonstrated a lower flux decline and irreversible fouling. Tian et al. [7] developed organic–inorganic hybrid membrane by blending various silane compounds with PVDF and hydrochloric acid as a catalyst. It was observed that the silane compounds created artificial ion channels within the nanocomposite membranes, hence improving the cation separation performance of the membranes. 

Forward osmosis (FO) has become a new tool for treating various wastewaters and for desalination application. The performance of FO processes greatly depends on the characteristics of the membranes. Suzaimi et al. [8] provided a timely overview on the strategies used for FO thin-film, composite-membrane substrate fabrication and modification. The use of nanomaterials has been highlighted as one of the most versatile and effective strategies in improving substrate layer properties. The roles of novel fabrication and modification in improving water flux, rejection, and antifouling have been thoroughly discussed. A critical consideration of the design of nanocomposite membrane comprises the selection of a well-matched combination of nanomaterials and a polymeric host to cater to intended applications. Goh et al. [9] presented a review to acknowledge the importance of understanding the roles of nanomaterial dimensions in nanocomposite membrane development. The review looked into the selection criteria of nanomaterials from the perspective of their dimensions for the production of high-performance functional nanocomposite membranes for both liquid and gas separation. 

The findings and critical discussion from these nine contributions have evidenced the roles and importance of nanocomposite membranes as valuable tools to heighten the performances of various membrane-based separation processes. The flexibility in coupling a wide range of nanomaterials and conventionally used membrane materials offers resultant nanocomposites with high versatility. The authors of these contributions have delved into the important aspects of this topic and addressed many important fundamental questions. The findings have led to a better understanding of material design and the possibilities to fill the research gaps. It is envisioned that this Special Issue could illustrate some beneficial guidelines and future trends in the development of more exciting and high-performance nanocomposite membranes. 
